# Effects of white noise on preterm infants in the neonatal intensive care unit: A meta‐analysis of randomised controlled trials

**DOI:** 10.1002/nop2.2094

**Published:** 2024-01-17

**Authors:** Qing Zhang, Qiugui Huo, Peizhen Chen, Wenying Yao, Zhihong Ni

**Affiliations:** ^1^ Department of Neonatology Children's Hospital of Soochow University Soochow China; ^2^ Department of Nursing Children's Hospital of Soochow University Soochow China

**Keywords:** meta‐analysis, preterm infant, randomised controlled trials, white noise

## Abstract

**Aim:**

To critically assess the effects of white noise on the pain level, weight gain and vital signs (heart rate, respiratory rate and oxygen saturation) of preterm infants in neonatal intensive care units (NICUs).

**Design:**

A systematic review and meta‐analysis of randomised controlled trials (RCTs).

**Methods:**

Ten databases (PubMed, Cochrane Library, Embase, Web of Science, CINAHL, PsycINFO, SinoMed, China National Knowledge Infrastructure, VIP and Wanfang Data) were systematically reviewed from inception to July 2022. Two reviewers evaluated the risk of bias separately using the Cochrane Collaboration criteria and extracted data using a predesigned information form.

**Results:**

The meta‐analysis included eight eligible RCTs. According to statistical analysis, white noise significantly affected the pain level, weight gain, heart rate, respiratory rate and oxygen saturation in preterm infants. Regardless of the outcome measurement timing, gestational age and birth weight of preterm infants, subgroup analysis demonstrated that white noise reduced the pain level, heart rate and respiratory rate and promoted weight gain in preterm infants in NICUs.

**Conclusion:**

White noise is a practical and potentially useful therapy for premature neonates in NICUs. No Patient or Public Contribution.

## 1 INTRODUCTION

Preterm infants have a gestational age of less than 37 weeks, with a birth rate of 5%–18% (Kelly & Tobias, 2021; Kobus et al., 2021). Due to limited intrauterine nutrition reserves, preterm infants, particularly those hospitalised in the neonatal critical care unit (NICU), have difficulty adapting to the exterior environment (Ryckman et al., 2017). They are exposed to numerous stresses, including high‐intensity sounds from medical equipment, constant bright light, painful iterative procedures, maternal separation and other stressors (Givrad et al., 2021; Mörelius et al., 2016; Vitale et al., 2021). As a result of relatively excessive stimuli, preterm infants are susceptible to adverse conditions, such as bradycardia or tachycardia, apnoea, hypoxemia, alterations in oxygen saturation and increased oxygen consumption. These can potentially decrease the number of calories available for growth (Almadhoob & Ohlsson, 2020; Cheong et al., 2020; Zhao et al., 2022). Thus, effective and proactive interventions are urgently required to prevent the negative effects of the above‐mentioned stressors on preterm infants.

Developmentally supportive care (DSC) is a comprehensive and conducive intervention program with such effects. It refers to providing supportive measures by improving the environment and nursing practices in the NICU to promote the normal and healthy growth of premature infants (Austin et al., 2019; Sathish et al., 2019). Under the DSC framework, various environmental management and non‐pharmacological interventions have been introduced in the NICU to minimise stress and enhance growth in preterm infants (Lee et al., 2022). Among them, auditory stimulations, such as white noise, maternal voice and a lullaby, are essential. White noise is a continuous, monotonous, resonant sound that is consistently dispersed throughout a wide frequency range, from 20 to 20,000 Hz (Riedy et al., 2021) and is portrayed as natural sounds such as wind, rain, and waves (Sundstrom et al., 1994). White noise resembles the sounds heard in the womb (Cetinkaya et al., 2022), where the infant is actively affected by the mother's heartbeat. After birth, exposure to these familiar noises and rhythms calms the infant.

A growing body of evidence revealed that white noise could draw preterm infants' attention away from painful stimuli because it blocks the unpleasant and irregular noise generated from the external environment, thereby significantly reducing the pain intensity (Lu et al., 2019). In addition, the expected benefits of white noise for preterm infants include a stable heart rate, improved respiratory rate, oxygen saturation, pacification, relaxation and growth promotion (Duan et al., 2017; Kahraman et al., 2020). From the aforementioned perspective, compared with the unpleasant auditory exposure in the NICU, white noise is a beneficial auditory stimulus and a stress reducer in preterm infants. On the other hand, several previous studies found no statistically significant differences between the experimental and control groups in terms of the reactions to the physiological stress imposed on preterm infants, such as their heart rates and oxygen saturation levels (Liao et al., 2021).

Jun et al. (2021) conducted a systematic review of clinical trials to investigate the impact of non‐pharmacological therapies employing white noise on the quality of critically sick adult patients in intensive care units. To our knowledge, however, there is no systematic review or meta‐analysis of the effects of white noise on preterm infants in general. In light of the benefits of white noise for preterm infants in the NICU and numerous accurate clinical trials completed in recent years on this topic, we decided to evaluate the effects of white noise on preterm infants in the NICU setting. The purpose of this study was to conduct a meta‐analysis of randomised controlled trials (RCTs) on the effect of white noise on pain level, weight gain and vital signs (including oxygen saturation, heart rate and respiratory rate) in preterm infants in the NICU.

## 2 METHODS

### 2.1 Design

This meta‐analysis adhered to the Cochrane Handbook for Systematic Reviews of Interventions guidelines and the updated Preferred Reporting Items for Systematic Review (Higgins et al., 2011) and Meta‐Analyses (PRISMA) guidelines (Page et al., 2021).

### 2.2 Search strategy and study selection

We searched the following 10 medical databases from inception to July 2022: PubMed, Cochrane Library, Embase, Web of Science, CINAHL, PsycINFO, China Biology Medicine (CBM), China National Knowledge Infrastructure (CNKI), VIP and Wanfang databases. We limited our search to RCTs published in English or Chinese, but there were no regional restrictions. The search terms used in a combined manner were (‘preterm infant’ or ‘premature infant’ or ‘low birth weight infant’ or ‘very low birth weight infant’ or ‘neonatal prematurity’ or ‘newborn’ or ‘neonate’) and (‘white noise’ or ‘acoustic stimulation’ or ‘sensory stimulation’ or ‘auditory intervention’ or ‘auditory stimulation’). In addition, relevant studies and grey literature were identified by manually searching the reference lists of the retrieved publications using Google.

Our overall study selection process was as follows: we initially imported the literature retrieved from 10 databases into EndNote X9 to find duplicates. Two reviewers (Q.Z. and Q.H.) then screened the titles and abstracts of the retrieved literature according to the inclusion and exclusion criteria for preliminary screening. Finally, the two reviewers (Q.Z. and Q.H.) independently read the full texts of the preliminarily screened literature and determined whether to include them in the study. Any disagreement was resolved through consensus between the two reviewers, and if consensus was not reached, it was resolved by the senior authors (W.Y. and Z.N.).

### 2.3 Inclusion and exclusion criteria

Studies that met the following PICOS (participants, intervention, comparator, outcomes and study design) eligibility criteria were included: (1) participants were preterm infants whose gestational age was less than 37 weeks and who were admitted to the NICU; (2) the intervention was white noise, with no limit to the timing, frequency and duration of the intervention; (3) studies in which individuals in the control group did not receive white noise were used for comparisons; (4) outcomes were subjectively assessed pain level measured using a pain scale by one or more medical personnel, weight gain measured using an electronic weighing machine and vital signs (heart rate, respiratory rate or oxygen saturation) measured using a monitor and (5) the study design included only RCTs.

The exclusion criteria were (1) studies with participants who did not pass the hearing test, (2) studies that used a combination of white noise and other interventions, (3) studies that did not evaluate or report the outcomes of interest, (4) studies in which the original text could not be verified, (5) studies with repetitive published literature, (6) letters to the editor, reports, conference abstracts, study protocols or qualitative studies and (7) studies that were not authored in English or Chinese.

### 2.4 Data extraction

Two reviewers (Q.Z. and Q.H.) separately examined the literature and extracted data using a pre‐designed data extraction form. The retrieved data included general information about the study (first author's name, publication year and country), sample size, participants' characteristics (gestational age and birth weight), intervention descriptions for experimental and control groups, outcome indicators and instruments. The senior authors resolved any disagreements (W.Y. and Z.N.).

### 2.5 Risk of bias assessment

The Cochrane Handbook for Systematic Reviews of Interventions, version 5.1.0 was used to evaluate the quality of each eligible study (Higgins et al., 2011), including the following six items: selection bias, performance bias, detection bias, attrition bias, reporting bias and other biases. Items in the risk of bias assessment were ‘low risk’, ‘high risk’ or ‘unclear’. In addition, the overall risk of bias was determined by ‘A' (all items had a low risk of bias), ‘B' (at least one item had an unclear risk of bias) or ‘C' (at least one item had a high risk of bias). Two reviewers (Q.Z. and Q.H.) assessed the risk of bias in the included studies independently and discussed any differences. The senior authors arbitrated any differences between the two reviewers (W.Y. and Z.N.). The Review Manager software (RevMan web version 5.3, The Cochrane Collaboration, available at revman.cochrane.org) was used to generate figures of potential biases within and across the included studies.

### 2.6 Data synthesis

Review Manager version 5.3 quantitatively synthesised the study findings relating to the effects of white noise on the outcomes of interest. Heterogeneity was determined using the chi‐squared test and *I* ^2^ statistics of the pooled data (Higgins & Thompson, 2002). When significant heterogeneity in the results was identified, that is, *p* < 0.1 and *I* ^2^ > 50%, a random effects model was used (DerSimonian & Kacker, 2007); otherwise, a fixed effects model was adopted (Mantel & Haenszel, 1959). When data heterogeneity was identified, subgroup analysis was conducted if at least two studies were within a stratum, as these characteristics might influence the results (Stevens et al., 2016). We used the weighted mean difference (WMD) with 95% confidence intervals (CIs) for continuous outcome measures. In contrast, the standardised mean difference (SMD) was selected when several outcome assessments for the same variable were utilised. With regard to interpreting the meta‐analysis results, an SMD value of 0.2, 0.5 or 0.8 indicated a small, moderate or large effect size of the intervention, respectively (Wu et al., 2021). Considering the stability of the results, a sensitivity analysis was performed by applying both random effect and fixed effect models and examining the relevant outcomes (Zhang et al., 2021). Since the meta‐analysis results included less than 10 studies, which was below the low test power level, we could not assess the publication bias (Song et al., 2002).

## 3 RESULTS

### 3.1 Study selection

**FIGURE 1:**
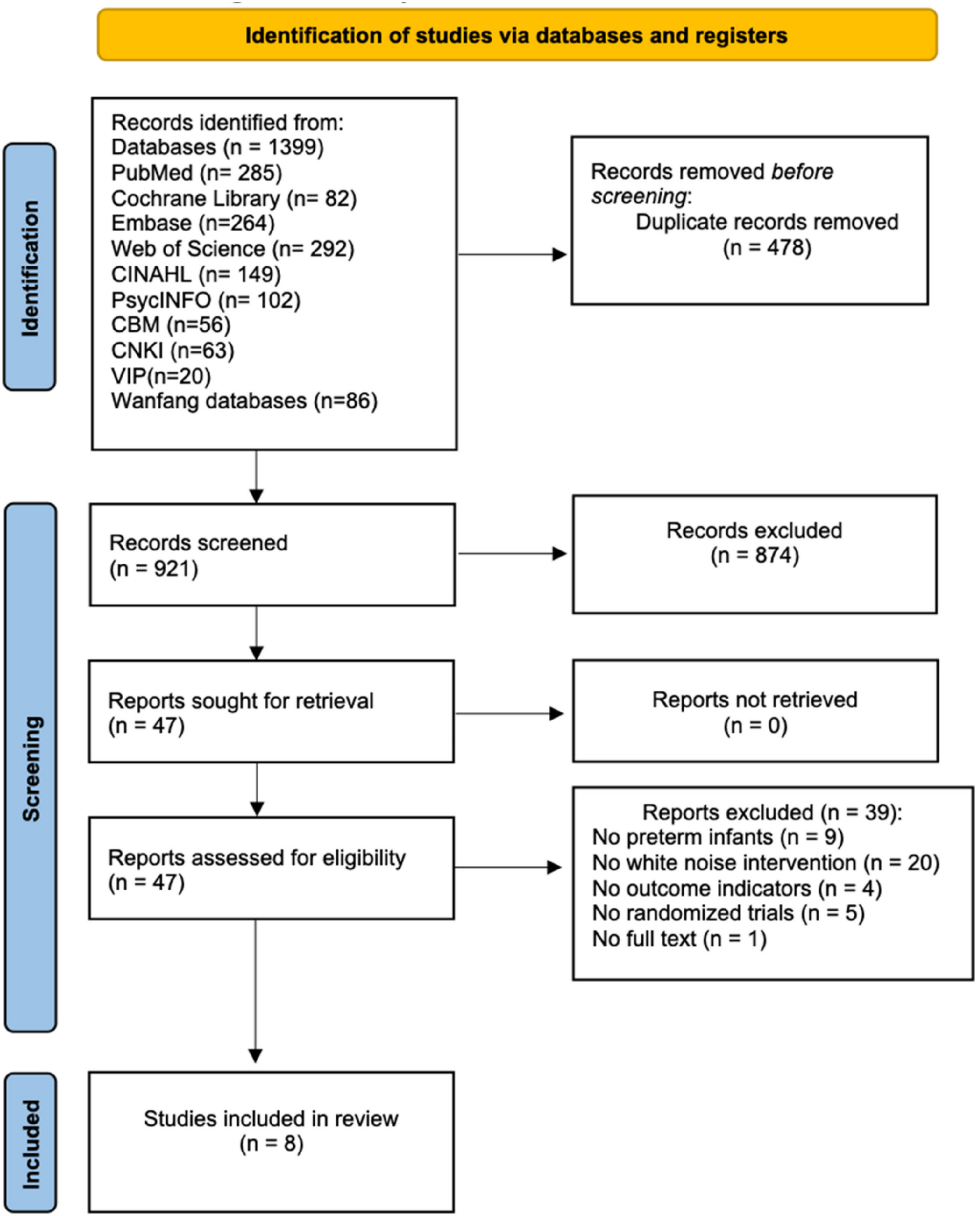
PRISMA flow diagram.

A PRISMA flow diagram is illustrated in Figure 1. A total of 1399 potentially eligible studies were identified using our search strategy for each database. In total, 478 duplicates were excluded. After reviewing the study titles and abstracts, 47 articles remained. Upon critical review of full texts, 39 articles were excluded (9 articles with participants who were not preterm infants in the NICU, 20 with no white noise interventions, 4 without outcome indicators, 5 that were not RCTs and 1 with no full text provided). Eight studies were finally included in the meta‐analysis (Döra & Büyük, 2021; Kahraman et al., 2020; Kucukoglu et al., 2016; Liao, 2018; Liao et al., 2021; Ren et al., 2019, 2021; Taplak & Bayat, 2021).

### 3.2 Characteristics of included studies

Table 1 presents the characteristics of the selected studies. A total of eight articles were included in this study—seven (Döra & Büyük, 2021; Kahraman et al., 2020; Kucukoglu et al., 2016; Liao et al., 2021; Ren et al., 2019, 2021; Taplak & Bayat, 2021) of which were published in academic journals, and one (Liao, 2018) was a master's thesis. Four studies were conducted in Turkey (Döra & Büyük, 2021; Kahraman et al., 2020; Kucukoglu et al., 2016; Taplak & Bayat, 2021), whereas the remaining were conducted in China (Liao, 2018; Liao et al., 2021; Ren et al., 2019, 2021). All eight studies were recently published RCTs, of which four were published in 2021 (Döra & Büyük, 2021; Liao et al., 2021; Ren et al., 2021; Taplak & Bayat, 2021), one in 2020 (Kahraman et al., 2020), one in 2019 (Ren et al., 2019), one in 2018 (Liao, 2018) and another in 2016 (Kucukoglu et al., 2016). The sample size ranged from 48 (Kahraman et al., 2020) to 396 (Ren et al., 2019), and in five of the eight studies, the sample size was less than 100 (Döra & Büyük, 2021; Kahraman et al., 2020; Kucukoglu et al., 2016; Liao, 2018; Taplak & Bayat, 2021). The gestational age of the participants ranged from 26 (Taplak & Bayat, 2021) to 37 weeks, and the maximum reported birth weight exceeded 1000 g (Song et al., 2002). Regarding white noise interventions in the experimental group, three studies selected the album ‘colic' to play (Kucukoglu et al., 2016; Ren et al., 2019, 2021), two selected ‘sound of light rain’ (Liao, 2018; Liao et al., 2021), one selected the track ‘don't let your baby cry’ (Döra & Büyük, 2021), one selected ‘the happiest baby’ (Kahraman et al., 2020), and one selected white noise based on expert opinion (Taplak & Bayat, 2021). Six studies controlled the playback volume of white noise at 50–55 dB (Kahraman et al., 2020; Kucukoglu et al., 2016; Liao, 2018; Liao et al., 2021; Ren et al., 2019, 2021), one controlled below 45 dB (Taplak & Bayat, 2021), and one played white noise 30–45 cm away from the preterm infants, and the specific volume was not clarified (Döra & Büyük, 2021). Six studies implemented white noise prior to and throughout unpleasant treatments (Döra & Büyük, 2021; Kahraman et al., 2020; Kucukoglu et al., 2016; Ren et al., 2019, 2021; Taplak & Bayat, 2021). In contrast, the remaining two studies implemented white noise before the feeding time (Liao, 2018; Liao et al., 2021). Of the eight included studies, only two described the frequency of white noise intervention (Liao, 2018; Liao et al., 2021), while the remaining six studies did not specify it (Döra & Büyük, 2021; Kahraman et al., 2020; Kucukoglu et al., 2016; Ren et al., 2019, 2021; Taplak & Bayat, 2021). All the studies included a control group that received either routine care (Kahraman et al., 2020; Liao, 2018; Liao et al., 2021; Ren et al., 2019, 2021) or underwent painful procedures without intervention (Döra & Büyük, 2021; Kucukoglu et al., 2016; Taplak & Bayat, 2021). All eight articles included in this study reported the heart rate and oxygen saturation of infants, whereas six reported pain levels (Döra & Büyük, 2021; Kahraman et al., 2020; Kucukoglu et al., 2016; Ren et al., 2019, 2021; Taplak & Bayat, 2021), two reported weight gain (Liao, 2018; Liao et al., 2021), and two reported respiratory rates (Liao, 2018; Liao et al., 2021).

**TABLE 1:** Characteristics of the included studies.

Author (year, country)	Sample size(n)	Gestational age(w) mean(SD)/median(IQR)	Birth weight(g) mean(SD)	EG intervention	CG intervention	Indicator (instrument)
Döra and Büyük (2021), Turkey	EG1: 22 EG2: 22 CG: 22	32–37 The specific values of each group are not clear.	>1000 The specific values of each group are not clear.	EG1: white noise (the track ‘Don't Let Your Baby Cry’ from Orhan Osman's album Kolik), 2 min before the routine blood collection and continued during and after the procedure; 30–45 cm away from the infants. EG2: Lullaby (lullaby selection was based on expert opinions); 2 min before the routine blood collection and continued during and after the procedure; 30–45 cm away from the infants.	Without intervention during routine blood collection.	Pain (PIPP) Heart rate Respiratory rate Oxygen saturation
Kahraman et al. (2020), Turkey	EG1: 20 EG2: 20 EG3: 20 CG: 20	EG1: 33.8 (1.75) EG2: 34.0 (1.50) EG3: 34.06 (1.76) CG: 34.25 (1.65)	EG1: 1909 (340) EG2: 1904 (325) EG3: 2186 (621) CG: 2201 (615)	EG1: white noise (‘the happiest baby’ by Dr. Harvery Karp); 5 min before the heel lance and during the procedure; 50 dB. EG2: recorded mothers' voice; 5 min before the heel lance and during the procedure; 50 dB. EG3: MiniMuffs; 5 min before the heel lance and during the procedure.	Routine care	Pain (NIPS) Heart rate Oxygen saturation
Kucukoglu et al. (2016), Turkey	EG: 35 CG: 40	EG: 31.77 (3.30) CG: 31.30 (2.50)	EG: 1673.29 (321.16) CG: 1530.62 (347.25)	EG: white noise (the album ‘Colic', by Orhan Osman of the On Music Production Company); 1 min before the vaccination and continued until 1 min after the procedure; 55 dB.	Without intervention during the vaccination.	Pain (PIPP) Heart rate Oxygen saturation
Liao (2018), China	EG1: 30 EG2: 31 CG: 30	EG1: 31 (30.5, 32.5) EG2: 30.7 (30, 33) CG: 32 (30.2, 33.1)	EG1: 1460 (230) EG2: 1430 (210) CG: 1510 (190)	EG1: white noise (sound of light rain); 20 min/time, 3 times/day, for 5 consecutive days; 40 min before the feeding time; 50–55 dB. EG2: mothers' humming (recorded mothers' singing of ‘Sleep, Sleep, My Dear Baby’ by Schubert, or ‘sleep, dear baby’ by Yuanzhe Zhang and Yemei Liu); 20 min/time, 3 times/day, for 5 consecutive days; performed 40 min before the feeding time; 50–55 dB.	routine care	Weight gain Heart rate Oxygen saturation
Liao et al. (2021), China	EG1: 34 EG2: 34 CG: 35	EG1: 31.4 (1.3) EG2: 31.3 (1.8) CG: 31.8 (1.7)	EG1: 1460 (220) EG2: 1430 (200) CG: 1530 (200)	EG1: White noise (sound of light rain); 20 min/time, 3 times/d, for 4 consecutive days; 40 min before the infant's scheduled care and feeding time; 50–55 dB. EG2: Mothers' voice (recorded mothers' singing of the Chinese version of lullaby); 20 min/time, 3 times/d, for 4 consecutive days; performed 40 min before the infant's scheduled care and feeding time; 50–55 dB.	routine care	Weight gain Heart rate Oxygen saturation
Ren et al. (2019), China	EG1: 96 EG2: 98 EG3: 102 CG: 100	EG1: 30.6 (1.3) EG2: 31.3 (1.2) EG3: 31.0 (1.4) CG: 31.2 (1.3)	EG1: 1460 (229) EG2: 1428 (268) EG3: 1476 (278) CG: 1518 (279)	EG1: white noise (the album ‘Colic', by Orhan Osman of the On Music Production Company); 1 min before the retinopathy screening and continued until 5 min after the procedure; 55 dB. EG2: Glucose. EG3: White noise combined with glucose.	Routine care	Pain (PIPP) Heart rate Oxygen saturation
Ren et al. (2021), China	EG1: 56 EG2: 58 EG3: 62 CG: 60	EG1: 31.05 (1.24) EG2: 30.63 (1.23) EG3: 30.37 (1.35) CG: 30.42 (0.97)	EG1: 1430.98 (231.67) EG2: 1442.71 (279.82) EG3: 1447.58 (261.27) CG: 1537.42 (245.76)	EG1: White noise (the album ‘Colic', by Orhan Osman of the On Music Production Company); before the retinopathy screening and continued until the procedure; 50–55 dB. EG2: non‐nutritive sucking. EG3: white noise combined with non‐nutritive sucking.	Routine care	Pain (PIPP‐R) Heart rate Oxygen saturation
Taplak and Bayat (2021), Turkey	EG1: 20 EG2: 20 EG3: 20 CG: 20	Range from 26–37 The specific values of each group are unclear.	≥1000 The specific values of each group are not clear.	EG1: white noise (the selection of white noise played to preterm infants was made based on the opinions from experts); 5 min before the endotracheal suctioning and continued until 5 min after the procedure; ≤45 dB. EG2: breast milk smell. EG3: facilitated tucking.	Without any intervention during the endotracheal suctioning.	Pain (PIPP‐R) Heart rate Respiratory rate Oxygen saturation

Abbreviations: CG, Control Group; EG, Experimental Group; IQR, Interquartile Range; NIPS, Neonatal Infant Pain Scale; PIPP, Premature Infants Pain Profile; PIPP‐R, Premature Infants Pain Profile‐Revised.

### 3.3 Risk of bias assessment

**FIGURE 2:**
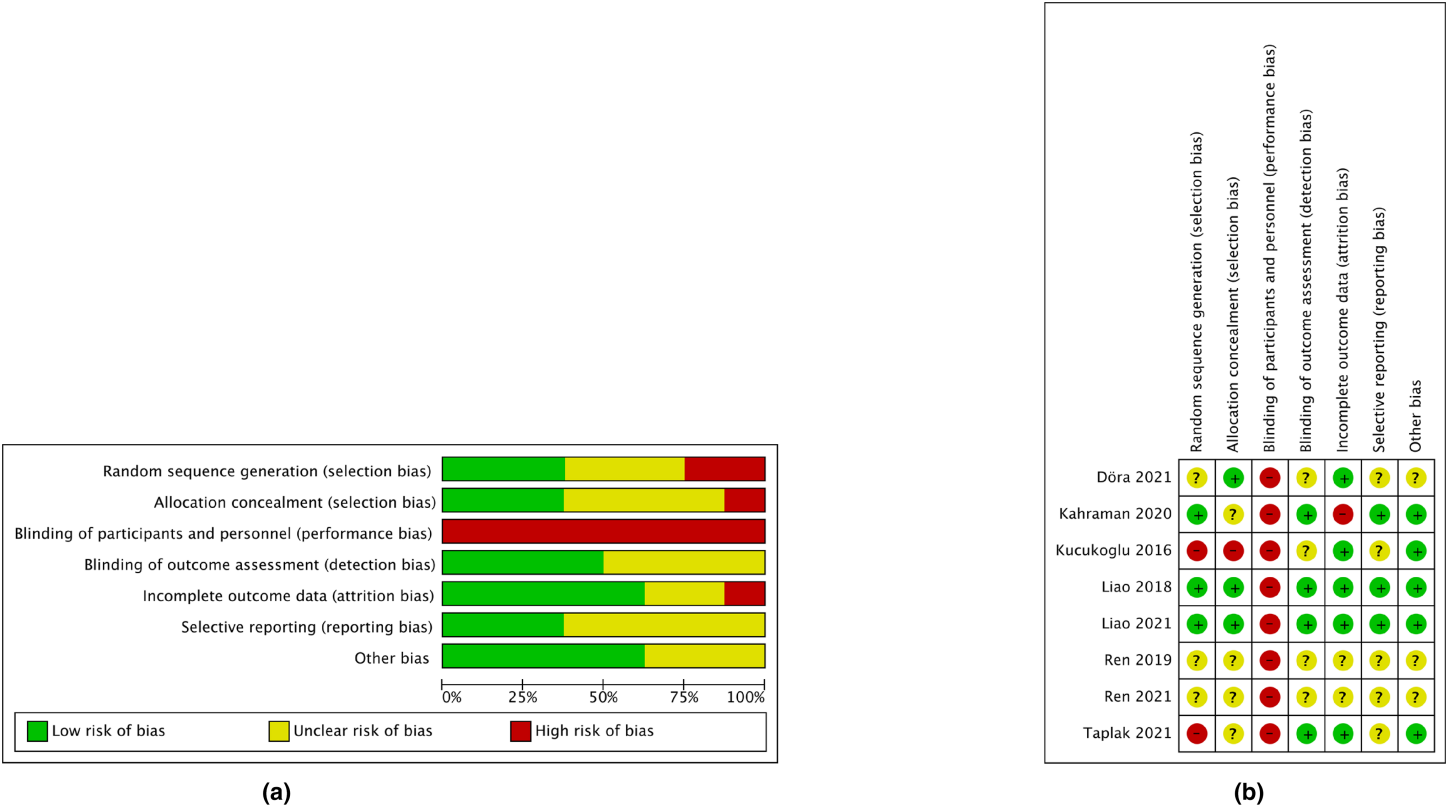
Risk of bias assessment using the Cochrane tool: (a) overall trials, (b) individual trials.

Regarding the performance bias, as blinding participants and personnel were inapplicable to the white noise intervention, we deemed this variable to be high risk for all studies. We mainly evaluated the blinding of the outcome assessment; four studies were at low risk (Kahraman et al., 2020; Liao, 2018; Liao et al., 2021; Taplak & Bayat, 2021) and the remaining four had an unclear risk (Döra & Büyük, 2021; Kucukoglu et al., 2016; Ren et al., 2019, 2021). Regarding the random sequence generation of the included studies, three studies did not mention specific methods (Döra & Büyük, 2021; Ren et al., 2019, 2021). Three studies reported allocation concealment (Döra & Büyük, 2021; Liao, 2018; Liao et al., 2021), one of which had a high risk (Kucukoglu et al., 2016). Concerning attrition bias, five studies had a low risk of bias (Döra & Büyük, 2021; Kucukoglu et al., 2016; Liao, 2018; Liao et al., 2021; Taplak & Bayat, 2021), two had an unclear risk (Ren et al., 2019, 2021) and one had a high risk (Kahraman et al., 2020). The protocols for the three studies were available (Kahraman et al., 2020; Liao, 2018; Liao et al., 2021), and these had a low risk of reporting bias; the risk of bias was unclear for the remaining five studies (Döra & Büyük, 2021; Kucukoglu et al., 2016; Ren et al., 2019, 2021; Taplak & Bayat, 2021). As we considered ‘blinding of participants and personnel’ to be high risk for all included studies, this item was not included in the assessment of overall study‐level risk of bias. Finally, two studies were graded ‘A' (Liao, 2018; Liao et al., 2021), three graded ‘B' (Döra & Büyük, 2021; Ren et al., 2019, 2021) and three graded ‘C' (Kahraman et al., 2020; Kucukoglu et al., 2016; Taplak & Bayat, 2021). Figure 2 depicts a summary of the bias risks for each domain across all studies.

### 3.4 The effects of white noise on preterm infants

#### 3.4.1 Pain level

**FIGURE 3:**
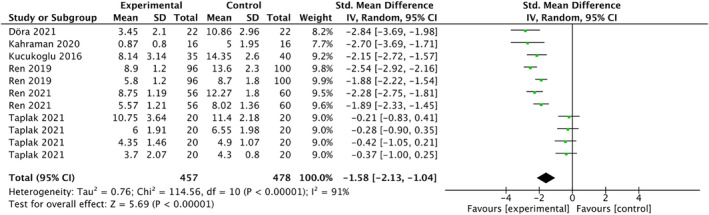
Forest plots for the effects of white noise on pain.

Forest plots in Figure 3 display the meta‐analysis results for the effects of white noise on pain levels. Data were obtained from a sample size of 935 participants (457 in the experimental group and 478 in the control group) from six studies (Döra & Büyük, 2021; Kahraman et al., 2020; Kucukoglu et al., 2016; Ren et al., 2019, 2021; Taplak & Bayat, 2021). Among the six studies included in this meta‐analysis, three measured pain using the Premature Infants Pain Profile (Döra & Büyük, 2021; Kucukoglu et al., 2016; Ren et al., 2019), two used the Premature Infants Pain Profile‐Revised (Ren et al., 2021; Taplak & Bayat, 2021) and one used the Neonatal Infant Pain Scale (Kahraman et al., 2020). Therefore, SMD was selected. The results indicated that white noise significantly affected the pain levels of preterm infants (SMD = −1.58; 95% CI: −2.13 to −1.04; *p* < 0.001). However, high heterogeneity was observed (*I* ^2^ = 91%).

#### 3.4.2 Weight gain

**FIGURE 4:**
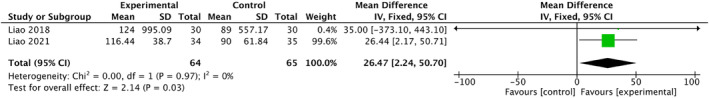
Forest plots for the effects of white noise on weight gain.

Forest plots in Figure 4 illustrate the meta‐analysis results of the effects of white noise on weight gain. Data from a sample size of 129 participants (64 in the experimental group and 65 in the control group) were obtained from two studies (Liao, 2018; Liao et al., 2021). The results indicated an enormous effect of white noise on weight gain in preterm infants (MD = 26.47; 95% CI: 2.24 to 50.70; *p* = 0.03). There was no heterogeneity identified among the studies (*I* ^2^ = 0%).

#### 3.4.3 Heart rate

**FIGURE 5:**
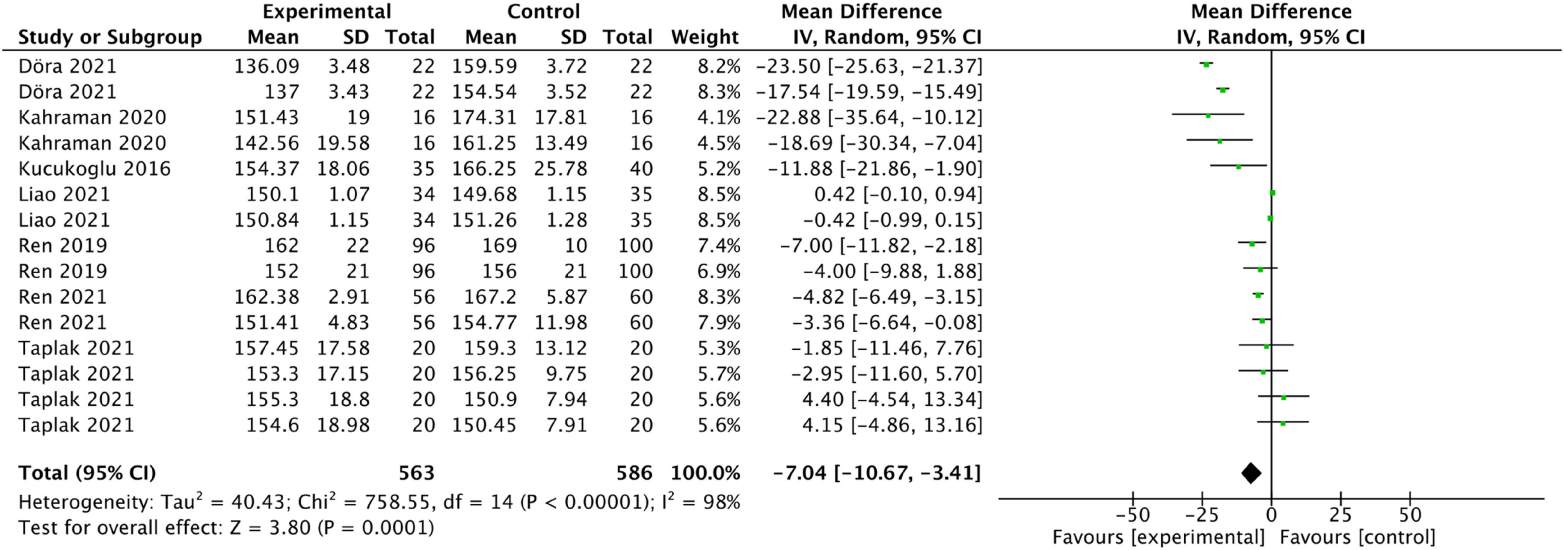
Forest plots for the effects of white noise on heart rate.

Forest plots in Figure 5 display the results of the meta‐analyses that reviewed the effects of white noise on the heart rate. All the included studies reported the heart rate, except one due to the lack of available data (Liao, 2018). Finally, data were obtained from 1149 participants (563 in the experimental group and 586 in the control group) in seven studies (Döra & Büyük, 2021; Kahraman et al., 2020; Kucukoglu et al., 2016; Liao et al., 2021; Ren et al., 2019, 2021; Taplak & Bayat, 2021). The results indicated that white noise significantly affected the heart rate of preterm infants (MD = −7.04; 95% CI: −10.67 to −3.41; *p* = 0.0001). However, high heterogeneity was observed (*I* ^2^ = 98%).

#### 3.4.4 Respiratory rate

**FIGURE 6:**
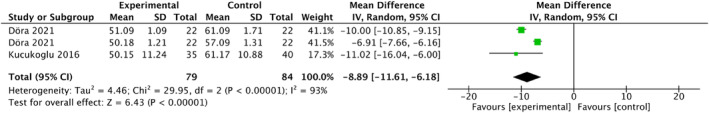
Forest plots for the effects of white noise on respiratory rate.

Forest plots in Figure 6 display the meta‐analysis results for the effects of white noise on the respiratory rate. Data from 163 participants (79 in the experimental group and 84 in the control group) were obtained from two studies (Döra & Büyük, 2021; Taplak & Bayat, 2021). The results indicated that white noise significantly affected the respiratory rate of preterm infants (MD = −8.89; 95% CI: −11.61 to −6.18; *p* < 0.0001). However, high heterogeneity was observed (*I* ^2^ = 93%).

#### 3.4.5 Oxygen saturation

**FIGURE 7:**
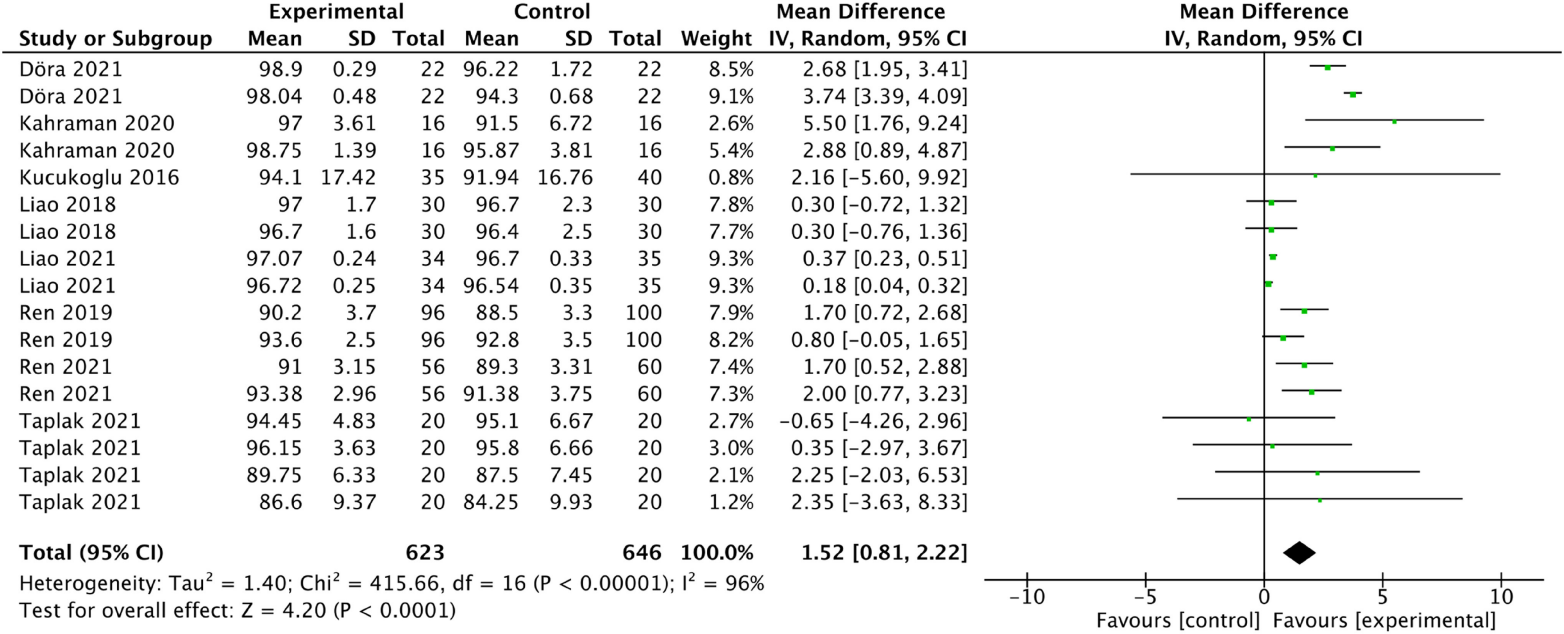
Forest plots for the effects of white noise on oxygen saturation.

Forest plots in Figure 7 illustrate the meta‐analysis results for the effects of white noise on oxygen saturation. Data from 1269 participants (623 in the experimental group and 646 in the control group) were obtained from eight studies (Döra & Büyük, 2021; Kahraman et al., 2020; Kucukoglu et al., 2016; Liao, 2018; Liao et al., 2021; Ren et al., 2019, 2021; Taplak & Bayat, 2021). The results indicated that white noise had a statistically significant effect on oxygen saturation in preterm infants (MD = 1.52; 95% CI: 0.81 to 2.22; *p* < 0.0001). However, high heterogeneity was observed (*I* ^2^ = 96%).

### 3.5 Subgroup and sensitivity analyses

Due to the substantial heterogeneity in the pain level, heart rate, respiratory rate and oxygen saturation, a subgroup analysis was required to determine its cause. No subgroup analysis was performed on the respiratory rate of preterm infants since there were insufficient data for this group. Subgroup analyses were performed by the measurement timing of the outcome, gestational age and birth weight of preterm infants as the modulating variables. Table 2 presents detailed information regarding the subgroups and sensitivity. According to the results of the sensitivity analysis, the model was relatively stable.

**TABLE 2:** Subgroup and sensitivity analysis.

Outcome indicators	Subgroups	*N*	Sample size		Random effect analysis			Fixed effect analysis		
			EG	CG	SMD/MD	95% CI	*p*‐value	SMD/MD	95% CI	*p*‐value
Pain level	Measure time									
	During the painful procedure	3	71	76	−1.66^a^	−3.15 to −0.17	0.03	−1.48^a^	−1.87 to −1.09	<0.0001
	After the painful procedure	4	386	402	−1.57^a^	−2.18 to −0.95	<0.0001	−1.77^a^	−1.94 to −1.60	<0.0001
	Gestational age									
	<32 weeks	3	339	360	−2.14^a^	−2.41 to −1.87	<0.0001	−2.13^a^	−2.32 to −1.95	<0.0001
	≥32 weeks	2	38	38	−2.78^a^	−3.43 to −2.13	<0.0001	−2.78^a^	−3.43 to −2.13	<0.0001
	Birth Weight									
	<1500 g	2	304	320	−2.14^a^	−2.47 to −1.81	<0.0001	−2.13^a^	−2.33 to −1.93	<0.0001
	≥1500 g	2	51	56	−2.29^a^	−2.78 to −1.79	<0.0001	−2.29^a^	−2.78 to −1.79	<0.0001
Heart rate	Measure time									
	During the painful procedure	3	58	58	−16.25^b^	−30.36 to −2.15	0.02	−22.50^b^	−24.55 to −20.45	<0.0001
	After the painful procedure	6	437	458	−6.18^b^	−11.18 to −1.17	0.02	−8.34^b^	−9.45 to −7.23	<0.0001
	Gestational age									
	<32 weeks	4	407	430	−2.57^b^	−4.28 to −0.86	0.003	−0.32^b^	−0.69 to 0.05	0.09
	≥32 weeks	2	76	76	−20.54^b^	−25.22 to −15.86	<0.0001	−20.42^b^	−21.88 to −18.96	<0.0001
	Birthweight									
	<1500 g	3	372	390	−2.28^b^	−3.95 to −0.60	0.008	−0.31^b^	−0.68 to 0.07	0.11
	≥1500 g	2	67	72	−16.88^b^	−23.40 to −10.36	<0.0001	−16.88^b^	−23.40 to −10.36	<0.0001
Oxygen saturation	Measure time									
	During the painful procedure	3	58	58	3.75^b^	3.40–4.09	<0.0001	3.75^b^	3.40 to 4.09	<0.0001
	After the painful procedure	6	437	458	1.77^b^	1.17–2.37	<0.0001	1.83^b^	1.47 to 2.24	<0.0001
	Gestational age									
	<32 weeks	5	467	490	0.60^b^	0.30–0.91	0.0001	0.32^b^	0.22 to 0.42	<0.0001
	≥32 weeks	2	76	76	3.32^b^	2.48–4.16	<0.0001	3.54^b^	3.23 to 3.85	<0.0001
	Birth Weight									
	<1500 g	4	432	450	0.61^b^	0.30–0.92	0.0001	0.32^b^	0.22 to 0.42	<0.0001
	≥1500 g	2	67	72	3.39^b^	1.68–5.11	0.0001	3.39^b^	1.68 to 5.11	0.0001

Abbreviations: CG, Control Group; EG, Experimental Group; MD, Mean difference; SMD, Standardised Mean Difference.

^a^ Applied SMD.

^b^ Applied MD.

#### 3.5.1 Measurement timing

Measurements of the outcomes at two different time points, that is, during and after the painful procedure, were analysed. The analysis revealed that white noise had a large effect on the pain level (SMD = −1.66; 95% CI: −3.15 to −0.17; *p* = 0.03), heart rate (MD = −16.25; 95% CI: −30.36 to −2.15; *p* = 0.02) and oxygen saturation (MD = 3.75; 95% CI: 3.40 to 4.09; *p* < 0.0001) of preterm infants during the painful procedure. Furthermore, white noise had a statistically significant effect on the pain level (SMD = −1.57; 95% CI: −2.18 to −0.95; *p* < 0.0001), heart rate (MD = −6.18; 95% CI: −11.18 to −1.17; *p* = 0.02) and oxygen saturation (MD = 1.77; 95% CI: 1.17 to 2.17; *p* < 0.0001) of preterm infants after the painful procedure.

#### 3.5.2 Gestational age

The gestational age classification was based on preterm babies being born before 32 weeks. Preterm infants with a gestational age of less than 32 weeks were very preterm infants, whereas those born at a gestational age of 32–37 weeks were moderate and late preterm infants (Lapillonne et al., 2019). One article (Taplak & Bayat, 2021) was excluded from further subgroup analysis because it did not provide the exact gestational age of the preterm infants. According to our findings, white noise had a significant effect on the pain level (SMD = −2.78; 95% CI: −3.43 to −2.13; *p* < 0.0001), heart rate (MD = −20.54; 95% CI: −25.22 to −15.86; *p* < 0.0001), and oxygen saturation (MD = 3.32; 95% CI: 2.48 to 4.16; *p* < 0.0001) in moderate and late preterm infants. Meanwhile, white noise significantly affected the pain level (SMD = −2.14; 95% CI: −2.41 to −1.87; *p* < 0.0001) and heart rate (MD = −2.57; 95% CI: −4.28 to −0.86; *p* = 0.003) in very preterm infants and had a moderate effect on oxygen saturation (MD = 0.60; 95% CI: 0.30 to 0.91; *p* = 0.0001) in very preterm infants. However, sensitivity analysis indicated no effect on the heart rate of very preterm infants (MD = −0.32; 95% CI: −0.69 to 0.05; *p* = 0.09).

#### 3.5.3 Birth weight

The birth weight classification was based on the weight of preterm infants at birth being less than 1500 g. Infants born prematurely with a birth weight of less than 1500 g have an extremely low birth weight (Eves et al., 2021). Two articles (Döra & Büyük, 2021; Taplak & Bayat, 2021) were excluded from further subgroup analysis because the exact birth weight of preterm infants in those articles was unclear. White noise had a statistically significant effect on the pain level (SMD = −2.29; 95% CI: −2.78 to −1.79; *p* < 0.0001), heart rate (MD = −16.88; 95% CI: −23.40 to −10.36; *p* < 0.0001), and oxygen saturation (MD = 3.39; 95% CI: 1.68 to 5.11; *p* = 0.0001) of preterm infants with a birth weight of at least 1500 g. In addition, the results suggested that in very low‐birthweight infants, white noise significantly affected the pain level (SMD = −2.14; 95% CI: −2.47 to −1.81; *p* < 0.0001) and heart rate (MD = −2.28; 95% CI: −3.95 to −0.60; *p* = 0.008) and had a moderate effect on oxygen saturation (MD = 0.61; 95% CI: 0.30 to 0.92; *p* = 0.0001). However, the sensitivity analysis demonstrated no effect on the heart rate (MD = −0.31; 95% CI: −0.68 to 0.07; *p* = 0.11).

### 3.6 Adverse effects

Only one study reported adverse effects, and no negative effects associated with white noise exposure were observed in this study (Liao et al., 2021).

## 4 DISCUSSION

To the best of our knowledge, this was the first study to conduct a meta‐analysis on the effects of white noise on weight gain, pain level and vital signs (heart rate, respiratory rate and oxygen saturation) in NICU preterm infants. This study confirmed that white noise has a positive effect on these outcomes. To better clarify the characteristics of the best intervention measures, the measurement timing of the outcomes, gestational age and birth weight of preterm infants was analysed in subgroups.

Weight gain is a marker of nutritional health in preterm infants and is independently associated with long‐term neurodevelopment (Skinner & Narchi, 2021). Due to an early departure from the superior living conditions of the womb and sudden changes in the growing environment outside the womb, preterm infants reportedly had a high energy consumption and a high incidence of various complications, which could directly affect the early weight gain rate (Patel & Rouster, 2022). In addition to medication supplementation with adequate nutrition, non‐pharmacological interventions can exert active effects to some extent (Lu et al., 2020; Thakkar et al., 2018). In the present study, white noise was useful in promoting weight gain in preterm infants. The underlying reason is that white noise soothes preterm infants, reduces their crying, and keeps them in a relaxed state, which not only reduces energy expenditure but also improves their ability to adapt to the extrauterine environment and thus helps increase their sucking ability (Ren & Yang, 2021; Zhang & He, 2022). However, since only two articles reported weight gain, both of which were from the same author and had small sample sizes, the effect of white noise on weight gain should be explored in further research.

Pain is a daily clinical exposure in the NICU environment and is regarded as the greatest risk factor for long‐term neurodevelopment in preterm infants (Hatfield et al., 2019; McPherson et al., 2020). Preterm infants in the NICU are subjected to invasive medical therapies on a regular basis in order to prevent, diagnose, and treat severe illnesses (Coviello et al., 2018). Furthermore, it was demonstrated that they were exposed to an average of 12–17 procedures daily in the NICU (Shiff et al., 2021). Therefore, identifying interventions that minimise pain levels with minor side effects is extremely urgent for healthcare providers and researchers. White noise masks noises for preterm infants, helping to neutralise negative environmental stimuli and creating a somewhat quiet and relaxed environment (Farokhnezhad Afshar et al., 2016), which assists in the modulation of pain. The results of this study confirmed that white noise reduces preterm infants' pain levels, regardless of the measurement timing of the outcomes, gestational age, and birth weight. Therefore, we recommend using white noise to relieve pain in preterm infants.

Our meta‐analysis revealed that white noise significantly stabilised vital signs in preterm infants, mainly manifested by an improved heart rate, respiratory rate, and oxygen saturation. This finding might be associated with the fact that white noise produces mild and benign stimulation of the preterm infants' hearing and vestibular system, which stimulates excitation of the limbic system in the brain, thereby relieving the physiological stress response and reducing nervous tension. It can make them feel safe and reduce their respiratory and heart rates. On the other hand, it can reduce oxygen consumption and increase the body's oxygen storage, thus improving oxygen saturation. Regarding the heart rate, subgroup analysis of the gestational age and birth weight of preterm infants indicated that white noise could significantly reduce the heart rate of very preterm infants and very low‐birthweight infants. However, sensitivity analysis revealed that this outcome was unstable. With respect to the respiratory rate, few studies were included. Due to these above‐mentioned factors, the results should be interpreted cautiously. Considering that white noise can reduce the heart and respiratory rates of preterm infants, more representative studies evaluating the above outcomes are needed in the future. In addition, subgroup analysis of the gestational age and birth weight of preterm infants indicated that the effects of white noise on increasing oxygen saturation of moderate and late preterm infants and those with a birth weight of at least 1500 g seemed better than those of very preterm and very low‐birthweight infants. This might be due to the poor hearing of very preterm and very low‐birthweight infants (Savenko et al., 2020), suggesting that this population might be less sensitive to white noise; thus, the effects of white noise on them were not statistically significant. Additionally, the small number of included studies may have influenced this conclusion; consequently, more studies should be added in future research.

Notably, adverse effects following the use of white noise are currently not a concern. However, infants may be excessively exposed to high levels of white noise, which can adversely impact the auditory pathway development and subsequent noise‐induced hearing loss (Hong et al., 2021). In this study, only Liao et al. (Liao et al., 2021) reported no negative effects from listening to white noise. Considering the small number of relevant studies, it may be premature to conclude whether white noise has a potent effect on neurodevelopment in preterm infants. Given the increasing prevalence of the use of white noise devices in preterm infants, further studies should include adverse effects as an outcome measure and clarify the results.

Of note, studies included in this study were from either Turkey or China, which might be because Turkey and China are both developing countries with many preterm births. In contrast, developed countries, have a smaller number of preterm births and higher allocation of human resources in the NICU, which means that the bed‐to‐care ratio in the NICU is lower. Preterm infants are more likely to receive timely soothing intervention in such settings and therefore have less need for white noise interventions. Thus, whether the results of this study are applicable to other countries needs to be further explored. Meanwhile, more empirical studies with more regions and larger sample sizes are required in the future to further verify the effects of white noise on preterm infants.

### 4.1 Limitations

This meta‐analysis is meaningful because it included RCTs and excluded other types of studies, such as quasi‐experimental studies. As such, the evidence was accurate and reliable. However, this study has some limitations to consider when interpreting the results. First, the results should be regarded with caution because the quality of the included articles was low, and the number of articles meeting the inclusion criteria in this study was small. Second, because this analysis only included papers published in English and Chinese, linguistic and reporting biases may have occurred. Third, there might have been a geographical limitation since the included studies were conducted only in China and Turkey. Fourth, due to the limitations of the original data, recommendations for some aspects of the white noise (duration and frequency of the white noise) could not be made. To provide more specific and detailed guidance for clinical applications, additional studies with a more robust scientific methodology using a multicentric design with larger sample sizes and evaluating the characteristics of white noise interventions are required.

## 5 CONCLUSION

White noise provided to preterm infants in the NICU can reduce pain levels, promote weight gain and stabilise vital signs, including the heart rate, respiratory rate and oxygen saturation. This finding implies that white noise can be used as a viable and successful therapy for preterm infants in the NICU. On the other hand, future studies should employ a more rigorous technique, including more studies with larger samples and careful consideration of the multiple features of white noise.

## AUTHOR CONTRIBUTIONS

Conceptualization: Q.Z., Z.N. and W.Y.; Methodology: Q.Z., Z.N. and W.Y.; Data curation: Q.Z. and Q.H.; Writing—the original draft: Q.Z.; Writing—reviewing and editing: Z.N., P.C. and W.Y. All authors have read and agreed to the published version of the manuscript.

## FUNDING INFORMATION

This work was financially supported by the Youth Science and Technology Project for Developing Healthcare through Science and Education, Suzhou, Jiangsu Province (KJXW2021022) and the Science and Technology Development Plan Project, Suzhou, Jiangsu Province (SKY2021048).

## CONFLICT OF INTEREST STATEMENT

All authors declare that they have no conflicts of interest.

## REGISTRATION

This review was not registered.

## RESEARCH ETHICS COMMITTEE APPROVAL

Not applicable.

## Data Availability

All data included in this review are available upon reasonable request by contacting the corresponding author.
